# Bladder cancer recurrence surveillance by urine metabolomics analysis

**DOI:** 10.1038/s41598-018-27538-3

**Published:** 2018-06-15

**Authors:** A. Loras, M. Trassierra, D. Sanjuan-Herráez, M. C. Martínez-Bisbal, J. V. Castell, G. Quintás, J. L. Ruiz-Cerdá

**Affiliations:** 1 Unidad Mixta de Investigación en Nanomedicina y Sensores, Universitat Politècnica de València – IIS Hospital La Fe, Valencia, Spain; 2 Servicio de Urología, Hospital Universitario y Politécnico La Fe, Valencia, Spain; 3 Health & Biomedicine, Leitat Technological Center, Barcelona, Spain; 4 Instituto Interuniversitario de Investigación de Reconocimiento Molecular y Desarrollo Tecnológico, Universitat Politècnica de València, Universitat de València, Valencia, Spain; 5 Departamento de Bioquímica y Biología Molecular, Universidad de Valencia, Valencia, Spain; 6 Unidad de Hepatología Experimental, CIBERehd, IIS Hospital La Fe, Valencia, Spain; 7 Analytical Unit, IIS Hospital La Fe, Valencia, Spain

## Abstract

Non Muscle Invasive Bladder Cancer (NMIBC) is among the most frequent malignant cancers worldwide. NMIBC is treated by transurethral resection of the bladder tumor (TURBT) and intravesical therapies, and has the highest recurrence rate among solid tumors. It requires a lifelong patient monitoring based on repeated cystoscopy and urinary cytology, both having drawbacks that include lack of sensitivity and specificity, invasiveness and care costs. We conducted an investigative clinical study to examine changes in the urinary metabolome of NMBIC patients before and after TURBT, as well during the subsequent surveillance period. Adjusting by prior probability of recurrence per risk, discriminant analysis of UPLC-MS metabolic profiles, displayed negative predictive values for *low*, *low-intermediate, high-intermediate* and *high risk* patient groups of 96.5%, 94.0%, 92.9% and 76.1% respectively. Detailed analysis of the metabolome revealed several candidate metabolites and perturbed phenylalanine, arginine, proline and tryptophan metabolisms as putative biomarkers. A pilot retrospective analysis of longitudinal trajectories of a BC metabolic biomarkers during post TURBT surveillance was carried out and the results give strong support for the clinical use of metabolomic profiling in assessing NMIBC recurrence.

## Introduction

Bladder cancer (BC) is the ninth most common cancer worldwide^1^. BC is associated with a high mortality rate (between 30 and 70% according to the disease stage) and the number of BC cases and deaths are expected to almost double in the near future because of smoking prevalence and the increase in life expectancy over time^1^. BC comprises two diseases entities with distinct clinical outcomes and very different molecular profiles: non-muscle invasive (NMIBC) and muscle invasive bladder cancer (MIBC)^2^. Despite its high prevalence and incidence, treatment of BC has not changed much over the past 25 years. MIBC is treated by radical cystectomy, adjuvant chemotherapy, or (chemo)-radiotherapy. On the other hand, NMIBC is treated by transurethral resection of the bladder tumor (TURBT) unspecific stimulation of the immune system (administration of Bacillus Calmette-Guerin, BCG) or intravesical chemotherapy (for example, mitomycin C)^3^.

NMIBC in the two early stages (Ta, non invasive papillary carcinoma; T_1_, tumor invades subepithelial connective tissue) represents 70–80% of the diagnosed cases and, although it is not life-threatening, it has the highest recurrence rate within tumors (up to 70% in five years^4^), requiring a lifelong monitoring of the patient after TURBT, by means of cystoscopy and urinary cytology. Active surveillance programs rely on the BC stage and grade at the time of detection. For high-grade tumors, cystoscopy and voided urine cytology are recommended every 3 months for 2 years, then every 6 months for the following 5 years and yearly thereafter. As each recurrence re-starts the monitorization schedule, patients are subjected to a very large number of cystoscopic examinations, making NMIBC one of the most health care demanding cancers. As an example, in the year 2010 bladder cancer was the 9^th^ most expensive cancer to treat and monitor in the USA, with cumulative costs of 4 billion US dollars, which represents 3.2% of all cancer-related care^5^.

BC diagnosis and therapy surveillance are largely based on cystoscopy and urinary cytology but both tests have important drawbacks, including lack of sensitivity and specificity, invasiveness and care costs^6^. Cystoscopy is the gold standard but it is certainly invasive, very uncomfortable for the patient, expensive and it can fail to notice certain lesions such as small areas of carcinoma *in situ* (CIS). Urine cytology is the most frequent non-invasive method used for the detection of BC. It has demonstrated its clinical utility when combined with cystoscopy, or when high-grade malignancy or CIS is present. However, cytological interpretation, although standardized by scales, relies on the ability of the examiner and affords a median sensitivity of 35% and median specificity of 94%^7^; it can only detect 50% of early stage/low-grade BC’s, just when they are most curable. The present limitations of cytology and cystoscopy have fostered the research on alternative, minimally invasive, approaches for BC diagnosis and surveillance.

The astonishing development of *omic’s* technologies in the past recent years combined with improved computing resources and mathematical models for data analysis, has expanded our capabilities for searching new biomarkers of disease. Precise genomic analysis, SNP’s epigenome analysis, transcriptomics, proteomics and more recently miRNA and metabolomic analysis have opened a vast field to search for new specific disease biomarkers. Nowadays, six urinary diagnostic tests for BC have been approved by the Food and Drug Administration (USA) for clinical use, namely, BTA stat, BTA TRAK, NMP22 BC, NMP22 BladderChek, uCyt+ and UroVysion. Despite initial promising results, these markers display higher sensitivity but lower specificity than urinary cytology, and unfortunately a low sensitivity for the early stages of BC recurrence compared to primary BC detection. As a matter of fact, no single urinary biomarker can replace cystoscopy – for the time being –as a non-invasive BC surveillance test.

The metabolome of a cell/tissue is the result of the interaction of the genome, epigenome, transcriptome, proteome and the set of external interventions. Metabolites are involved in almost every biochemical reaction in the human body including signaling pathways and hence the metabolome is considered to provide a direct meaningful readout of the dynamic biochemical status of a biological system. Because of that, metabolomics is now considered as a highly relevant approach to explore individual phenotypes in systems biology of cancer^4^. Differences in metabolism between cancer and normal cells are recognized as hallmarks of cancer^4^. Tumors reprogram pathways of nutrient acquisition and metabolism to meet bioenergetic, biosynthetic and redox demands^8^ as well as metabolic control of inflammation and immunity that differentiate benign from cancerous tissues. Metabolomic studies in BC have shown altered energy, cell membranes formation, nucleic acid synthesis and oxidative stress pathways biomarkers^9,10^. Consistent with these findings, preliminary studies point at using metabolic profiles with discriminatory capabilities for BC, as novel urinary biomarkers^11–18^.

BC is a pan-urothelial disease and the urinary metabolic profile should be considered as the expression of the tumor as well the entire urothelium. It is then conceivable that after TURBT, the metabolic profile may undergo changes approaching a *healthy* baseline profile. Thus, a study aiming at the identification of new diagnostic biomarkers of primary BC should include patients undergoing investigation for suspected bladder cancer and with other non-cancerous urothelium affectations. Conversely, the occurrence of disease biomarkers in the course of disease recurrence should include patients undergoing surveillance after TURBT. Previous studies aiming at the identification of metabolomic biomarkers for BC diagnosis were limited by the use of heterogeneous cohorts of NMIBC and MIBC patients, not fully representative of the target population^19^ and that difficulted the interpretation of the outcomes as multiple distinct molecular subtypes and/or individual phenotypes of MIBC and NMIBC^2^ were jointly analyzed.

In order to circumvent these problems, we conducted, to the best of our knowledge, the first clinical investigative study for the analysis of urinary metabolome changes in NMIBC patients before and after TURBT, as well in cancer recurrence, using ultraperformance liquid chromatograhy combined with time of flight mass spectrometry (UPLC-TOFMS). As results of our work, it has been possible to identify metabolites capable of discriminating BC patients with a high sensitivity (87.9%) and specificity (100%), and a negative likelihood value of 0.1, as well high negative predictive values for low, low-intermediate and high-intermediate and high-risk patients. The metabolomic analysis revealed altered phenylalanine, arginine, proline, and tryptophan intermediate metabolism associated to NMIBC. Results from analysis of longitudinal trajectories of the metabolic profile during surveillance after TURBT gave support to the idea of using a metabolomic approach to monitor early NMIBC recurrence in patients.

## Experimental Section

### Patients

The present study was approved by the Ethics Committee for Biomedical Research of the Instituto de Investigación Sanitaria Hospital Universitario y Politécnico La Fe (Valencia, Spain) (approval number 2012/0186) and all methods were performed in accordance with the relevant guidelines and regulations. Urine samples were prospectively collected from patients that had given written informed consent to participate in the study. Patients with diagnosed bladder tumor undergoing planned transurethral resection of the bladder tumor (TURBT) were invited to participate in the study. Inclusion criteria were: 20–90 years old males, NMIBC diagnosed, single or multiple tumors, tumor size greater than 0.5 cm, primary or recurrent tumors. No therapy was used at the time of sample collection. Exclusion criteria were: urinary catheter carrier, re-staging TURBT, rescue TURBT because of incomplete first TURBT, bladder randomized biopsy. Also, patients with infiltrative tumor diagnosed (pT2-4), no tumor diagnosed (pT0), papilloma or pTis pathological anatomy diagnosis, were excluded. After recurrent risk group classification according to the European Organisation for Research and Treatment of Cancer score, several patients were included in a monthly monitoring group to collect serial urine samples until recurrence. In this study, we analyzed the metabolomic profiles of 316 urine samples collected from 31 patients between March 2012 and December 2016. Table 1 summarizes the main features and pathological data of patients included in the study. Urine samples were collected from patients diagnosed with bladder cancer by cystoscopy and tissue pathology and coded as BC. Those collected from NMIBC patients within 2–4 weeks after TURBT were coded as CTRL. Urines collected after TURBT with negative cystoscopy at the time of sampling and those collected in the course of regular visits to the urologist between negative cystoscopies were classified as MONITOR. If no cystoscopy was available at the time of sampling or after, samples were classified as NA and were not included in the estimation of figures of merit.

**Table 1:** Demographic and clinical overview of recruited patients.

	Train set	Validation set
Patients (male/female)	18 (13/5)	28 (23/6)
Age (mean and standard deviation)	67 (11)	63 (8)
Samples (male/female)	53 (38/15)	210 (169/41)
**PLS-DA model BC** ***vs*** **CTRL**		
Samples pre-TURBT (BC)	35	33
Samples post-TURBT (CTRL)	18	11
Samples surveillance (MONITOR)	0	166*
Primary/Recurrent BC	8/27	7/23 (3: NA)
Tumor stage (pTx, pTa, pT1)	1/21/13	0/21/3 (9: NA)
Tumor grade (High/Low)	7/28	7/14/3 (9: NA)
**PLS-DA model BC** ***vs*** **MONITOR**		
Samples pre-TURBT (BC)	35	33
Samples post-TURBT (CTRL)	0	29*
Samples surveillance (MONITOR)	82	84
Primary/Recurrent BC	8/27	7/23 (3: NA)
Tumor stage (pTx, pTa, pT1)	1/21/13	0/21/3 (9: NA)
Tumor grade (High/Low)	7/28	4/22 (7: NA)
**PLS-DA model CTRL** ***vs*** **MONITOR**		
Samples pre-TURBT (BC)	0	68*
Samples post-TURBT (CTRL)	18	11
Samples surveillance (MONITOR)	82	84
Primary/Recurrent BC	0	15/50 (3: NA)
Tumor stage (pTx, pTa, pT1)	0	1/42/16 (9: NA)
Tumor grade (High/Low)	0	11/50 (7: NA)

Note: ^*^ indicates that these samples were not used for the estimation of the discriminant performance in that particular model.

### Sample preparation

Urine samples, once collected were kept at −80 °C until analysis. Samples were thaw at room temperature on ice, vortexed for 10 s and centrifuged at 10000 × *g* (4 °C, 10 min). Then, 100 µL of supernatant was withdrawn and 200 µL of HCOOH 0.1% v/v in H_2_O was added and the solution was vortexed 10 s and centrifuged at 10000 × *g* (4 °C, 10 min). No molecular weight cutoff (MWCO) filters were used during sample preparation. Thereafter, 100 µL of the supernatant was transferred to a 96 well plates where each sample was spiked with 5 µL of an internal standard solution containing Phenylalanine-D_5_ (Cambridge Isotopes Laboratory Inc., Andover, MA, USA), caffeine-D_9_ (Toronto Research Chemicals, Toronto, Ontario, Canada), leukine enkephalin (Sigma-Aldrich Química SA, Madrid, Spain) and reserpine (Sigma-Aldrich Química) in H_2_O:CH_3_OH (1:1, 0.1% v/v HCOOH), at a final concentration of 1 µM each. Control blanks were prepared by replacing urine by H_2_O. A quality control (QC) sample was prepared by mixing 5 µL of each prepared sample. All solvents were of LC-MS grade and were purchased from Scharlau (Barcelona, Spain). Ultra-pure water was generated with a Milli-Q water purification system (Merck Millipore, Darmstadt, Germany). Formic acid (≥95%) was obtained from Sigma-Aldrich Química.

### UPLC-TOF-MS sample analysis

Chromatographic analysis was performed on an Agilent 1290 Infinity UPLC chromatograph using a UPLC BEH C_18_ (100 × 2.1 mm, 1.7 µm, Waters, Wexford, Ireland) column. Autosampler and column temperatures were set to 4 °C and 55 °C, respectively and the injection volume was 4 µL. A gradient elution was performed at a flow rate of 400 µL min^−1^ as follows: initial conditions of 98% of mobile phase A (0.1% HCOOH in H_2_O, v/v) were kept for 0.5 min, followed by a linear gradient from 2% to 20% of mobile phase B (0.1% v/v HCOOH in CH_3_CN) for 3.5 min and from 20% to 95% B in 4 min. 95% B was held for 1 min and then, a 0.25 min gradient was used to return to the initial conditions. Between runs, the initial conditions were held for 2.75 min for column re-equilibration. Full scan MS data from 70 to 1700 m/z Da, with a scan frequency of 6 Hz (1274 transients/spectrum) was collected on a quadrupole time of flight (QTOF) Agilent 6550 spectrometer (Agilent Technologies, CA, USA) in the TOF MS mode. The following positive electrospray ionization (ESI) parameters were selected: gas T, 200 °C; drying gas, 14 l/min; nebulizer, 37 psig; sheath gas T, 350 °C; sheath gas flow, 11 l/min. Automatic MS spectra recalibration during analysis was carried out introducing a mass reference standard into the source via a reference sprayer valve using the 149.02332 (background contaminant), 121.050873 (Purine) and 922.009798 (HP-0921) m/z Da as references. Sample acquisition was randomized and the QC sample was analyzed every 5 injections to monitor and correct changes in the instrument response. Eight replicates of the QC were injected at the beginning of each batch for column conditioning. Data acquired during conditioning was excluded from the analysis. The sample set included 315 urine samples, 56 QCs, and 4 blanks. Sample analysis was carried out in two batches to reduce the time that samples are kept in the autosampler during analysis. Batch 1 included 224 injections of 187 urine samples (35 BC, 19 CTRL, 105 MONITOR, 28 NA), 4 Blanks and 33 QCs. Batch 2 included 152 injections of 129 urine samples (35 BC, 10 CTRL, 61 MONITOR, 23 NA) and 23 QCs. All samples from the same patient were analyzed in the same batch. Patient distribution between batches was randomized.

#### Peak table generation and data quality assessment

Centroid raw UPLC-TOF-MS data was converted into mzXML format using ProteoWizard (http://proteowizard.sourceforge.net/) before generating peak tables using XCMS software^20^. The centWave method was used for peak detection with the following parameters: ppm: 15, peakwidth: (5, 20), signal to noise threshold: 6. A minimum difference in m/z of 5 mDa was selected for peaks with overlapping retention times (RTs). Intensity weighted m/z values of each feature were calculated using the wMean function. Peak limits used for integration were found through descent on the Mexican hat filtered data. Peak grouping was carried out using the ‘nearest’ method using mzVsRT = 1 and RT and m/z tolerances of 6 s and 5 mDa, respectively. After peak grouping, the fillPeaks method with the default parameters was applied to fill missing peak data. RT and m/z tolerances used for peak table generation and alignment of features across batches was based on the observed variation in five selected metabolites (phenylalanine, tryptophan, kynurenine, hydroxykynurenine, and phenylacetylglutamine) and spiked internal standards (ISs) (phenylalanine-D_5_, caffeine-D_9_, leukine enkephalin and reserpine); see for example Fig. S1. Peak integration accuracy was assessed by comparing automated and manual integration results for internal standards (Fig. S1). A total of 4299 and 4416 LC-MS features found after peak detection, integration chromatographic de-convolution in batches 1 and 2, respectively identified by the m/z (Da) and retention time (min). Alignment of features led to 3226 LC-MS features. Blank samples were used to identify and remove background features arising from e.g. source contaminants, plasticizers, or solvent impurities. Within-batch effect elimination was performed by fitting time dependent non-linear functions to the injected QCs followed by a normalization of the data to this function using QC-SVRC and a radial basis function kernel, as described elsewhere^21^. The *ε*-insensitive loss parameter, the error penalty C and the kernel parameter *γ* used for the fitting of the SVR functions were selected using the 10-fold root mean squared cross validation error (RMSECV) as estimates of the expected generalization error. The ε-insensitive loss parameter for each metabolic feature was selected as the expected instrumental precision (i.e. ±2.5% of the median value observed in QCs). The error penalty C was calculated as the median value of the responses in QCs^21^. The kernel parameter *γ* providing the lowest RMSECV for each variable in the [2^−3^, 2^−2^, …, 2^9^] range was selected. Between-batch effects were eliminated by scaling the intensity of each metabolic feature in each sample using a factor defined as the ratio between the median intensity in QCs in the corresponding batch and the median intensity across batches. Finally, metabolic features showing RSD% >15 in QCs were considered unreliable and removed, leaving 2006 features for data analysis. Batch effects affecting the number of missing values due to e.g. instability of the chromatographic separation or wrong feature alignments were not considered in this work.

#### Chemometric and statistical analysis

The data set was initially split into two subsets for train and validation. The train set was used for partial least squares – discriminant analysis (PLS-DA) model development and feature selection. The validation set was exclusively used for the evaluation of the model predictive performance. Data scaling included multiplicative scatter correction with the median QC as reference followed by pareto scaling. The selection of the optimal number of PLS-DA latent variables (LVs) was carried out using the root mean square error of cross validation (RMSECV) and a leave-*one patient-*out CV strategy. The classification accuracy, the area under the receiver operating curve (AUROC) as well as the sensitivity, selectivity and negative and positive likelihood ratios were employed as PLS-DA figures of merit.

Identification of metabolites was carried out by matching m/z (Da) values against the Human Metabolome Database (HMDB, http://www.hmdb.ca) and METLIN databases (http://metlin.scripps.edu/) with 5 ppm accuracy. Molecular formulae were estimated by MassHunter Workstation Software-Qualitative Analysis (Agilent). Data acquisition and manual integration of peaks of IS and selected metabolites were carried out using MassHunter workstation (Agilent). PLS-DA was carried out using PLS Toolbox 8.0 (Eigenvector Research Inc., Wenatchee, USA) and in-house written MATLAB (Mathworks Inc., Natick, MA, USA) scripts. Support Vector Regression was carried out in MATLAB using the LIBSVM library^22^. Pathway analysis was carried out with MetaboAnalyst 3.0^23^. MATLAB scripts used in this work are available from the authors. The datasets generated during and/or analyzed during the current study are available from the corresponding author on reasonable request.

## Results and Discussion

### Data overview and quality assessment

The replicate analysis of a QC sample throughout the batch enables a straightforward evaluation of the instrument performance^24^. Under optimal conditions, technical variation should lead to random variation in intensities across QC replicates. However, the plot of the peak areas (AU, arbitrary units) in QCs as a function of the injection order showed trends both within and between batches, as well as heteroscedastic variance across batches (see Fig. S1). Likewise, cumulative distribution functions of the relative standard deviations in QCs (RSD_QC_) in raw data depicted in Fig. S2 showed a significantly better instrument performance in terms of repeatability (i.e. lower RSD_QC_) in batch 2. Figure S2 depicts PC1-PC3 scores of a PCA model for QC replicates as a function of the injection order, showing a significant between-batch effect in PC1 and within-batch effects in PC2 and PC3. Batch effects difficult the accurate identification of underlying trends in the data and so, an initial batch effect correction was carried out as described above. After, within- and between-batch effect correction, the number of metabolic features showing RSD_QC_ <15% increased from 652 up to 2006, and the median RSD_QC_ decreased from 20.2% down to 8.7%. PCA scores after batch effect correction depicted in Fig. S2 showed no association with the injection or batch order, in agreement with results depicted in Fig. S1 where the corrected intensities of the previous set of 5 metabolites as a function of the injection order are depicted. A PCA model of the set of BC, MONITOR and CTRL samples was calculated. The PC1 vs PC2 scores plots obtained from the PCA models of the set of BC, MONITOR, and CTRL samples after batch effect correction showed a high overlap of BC, MONITOR and CTRL samples (see Fig. S3). No clustering among the groups was observed using higher PCs (data not shown). PCA did not reveal a specific structure related to BC progression. Nonetheless, the PCA model was used to assess the absence of outlying samples based on their relative position to the 95% confidence limit.

### Discriminant analysis among BC, CTRL and MONITOR samples

**Figure 1:**
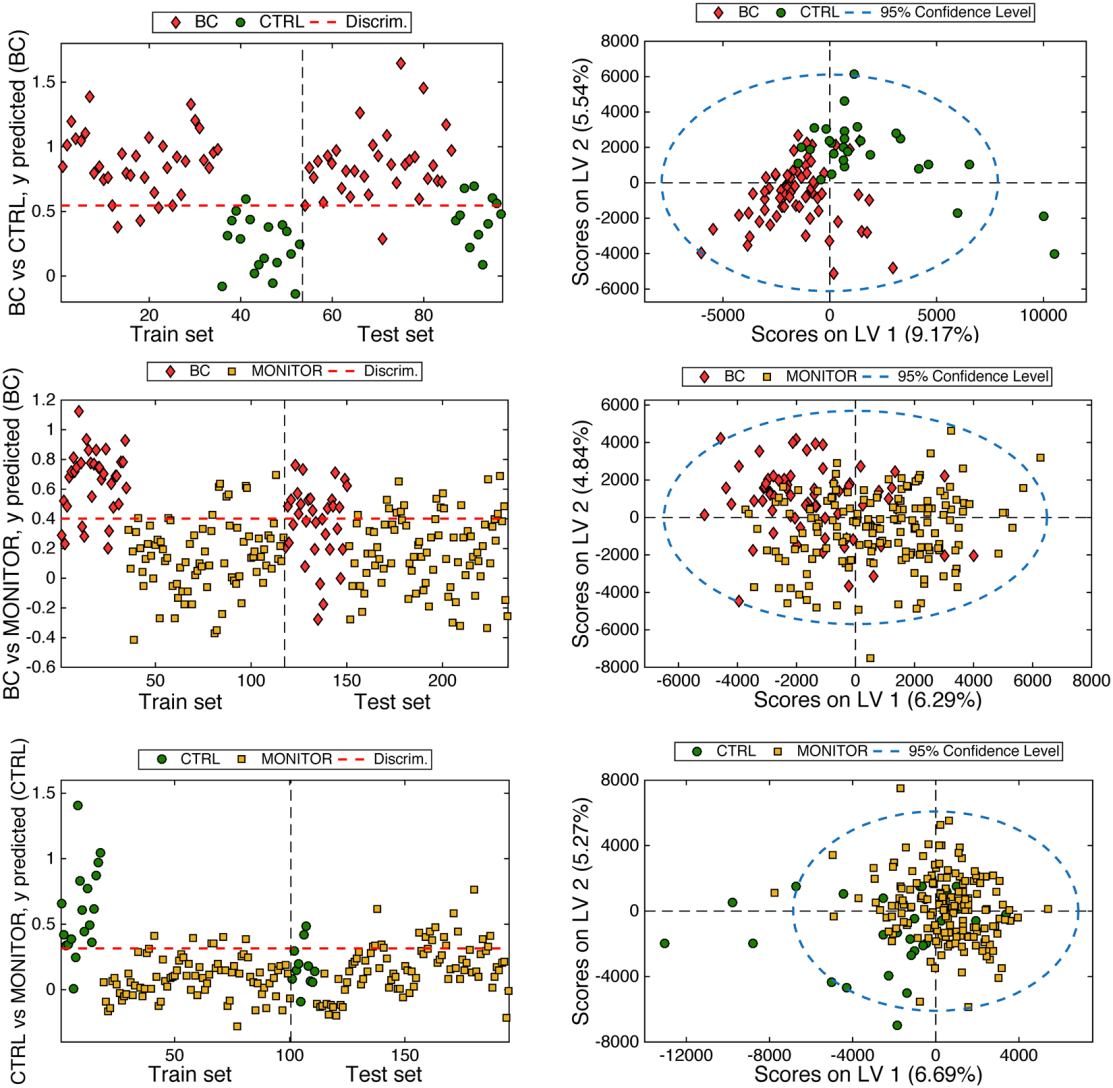
Discriminant analysis of BC, CTRL and MONITOR samples. (Left) PLS-DA predicted *y* values for the train (autoprediction) and test subsets; (Right) scores plot for the train and test sets. Number of LVs: 3.

To disclose the differences in the metabolic profiles among BC, CTRL and MONITOR samples, three independent PLS-DA models were considered in which the groups were pairwise compared (i.e. BC *vs* CTRL, BC *vs* MONITOR and CTRL *vs* MONITOR). Train and tests sets selected for the three models are summarized in Table 1. PLS-DA scores plots and predicted values for the three models depicted in Fig. 1 and the figures of merit calculated for the validation sets summarized in Table 2 showed a statistically significant shift in the urinary metabolic profiles after TURBT. The BC *vs* CTRL model provided an accurate sample classification of 27/33 BC and 10/11 CTRL (sensitivity: 82% and specificity: 91%). The second model, build for the discrimination between BC *vs* MONITOR samples, performed worse in classifying BC samples (sensitivity: 70% and specificity: 75%). Finally, the analysis of the differences between CTRL *vs* MONITOR groups provided non-significant predictive performances, lower sensitivity (45%) and specificity (76%) values, in agreement with the higher overlapping of CTRL and MONITOR samples, as depicted in Fig. 1.

**Table 2:** Indices of test validity estimated for the evaluation of the predictive performance of PLS-DA models between BC *vs* CTRL (LVs: 3), BC *vs* MONITOR (LVs: 3) and MONITOR *vs* CTRL (LVs: 3) samples in the validation set.

LVs	PLS-DA model		
	BC *vs* CTRL	BC *vs* MONITOR	CTRL *vs* MONITOR
	3	3	3
AUROC	0.94	0.75	0.53
Sensitivity	81.8 (64.5–93.0)%	69.7 (51.3–84.4)%	45.4 (16.7–76.6)%
Specificity	90.9 (58.7–99.8)%	75.0 (64.4–83.8)%	76.0 (66.6–83.8)%
PLR	9.0 (1–4–58.7)	2.8 (1.8–4.3)	1.9 (0.9–3.9)
NLR	0.2 (0.00–0.42)	0.4 (0.2–0.7)	0.7 (0.4–1.2)

PLR: positive likelihood ratio; NLR: negative likelihood ratio.

**Figure 2:**
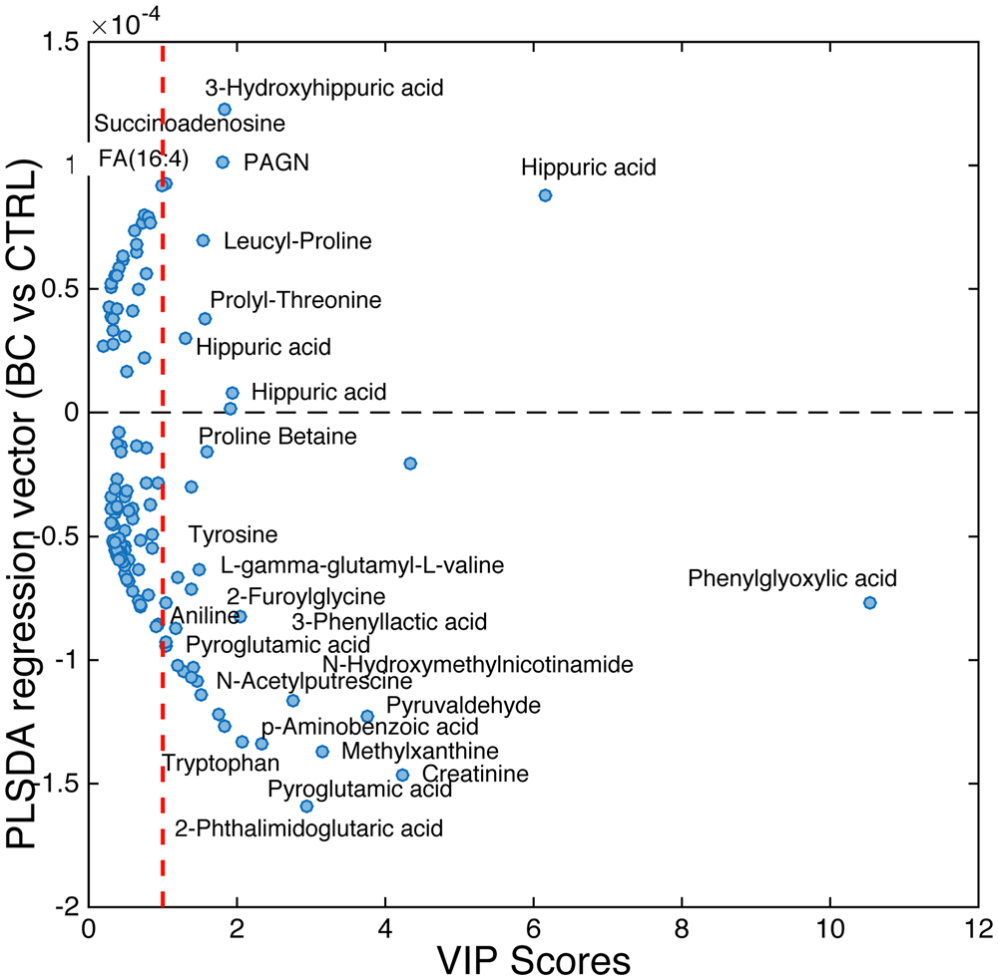
Discriminant metabolites between BC and CTRL samples. VIP scores as a function of the value in the PLS-DA regression vector in a model build using 128 selected metabolic features.

**Figure 3:**
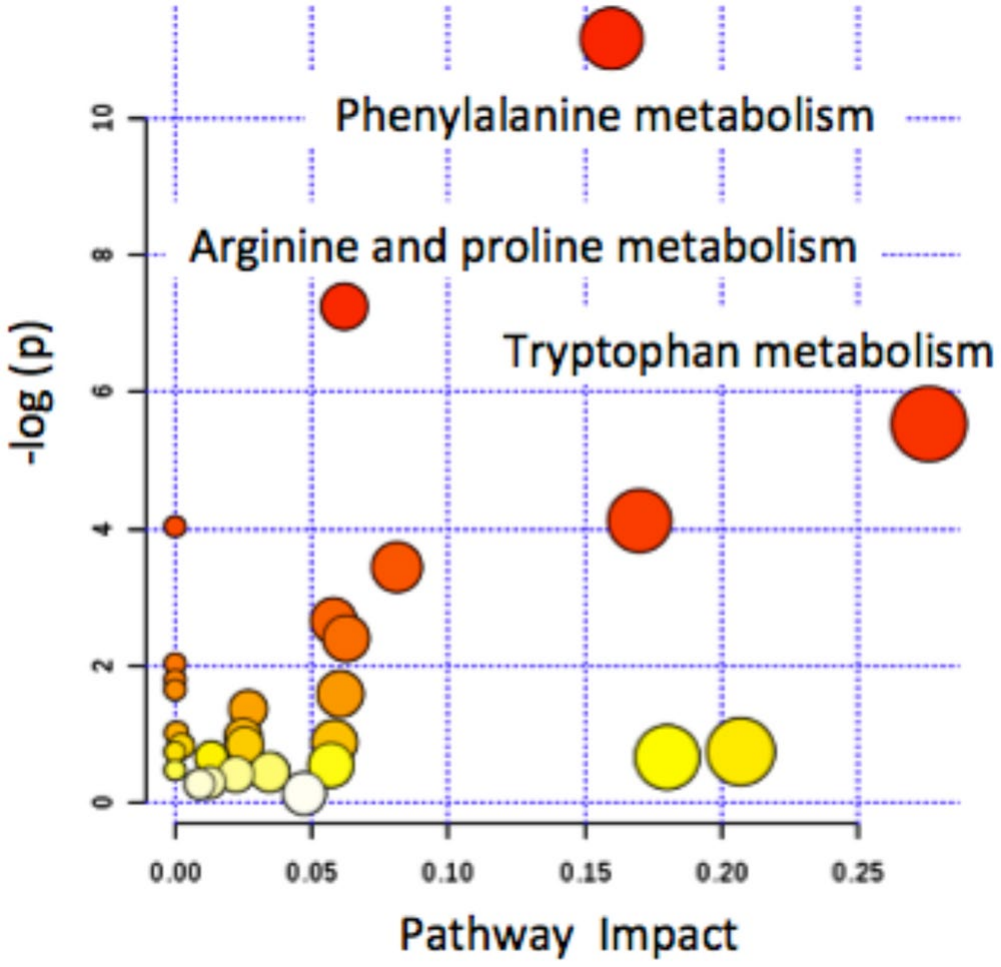
Pathway analysis of the urinary metabolic shift after TURBT. Results from pathway analysis, using a global test for enrichment analysis and a relative-betweeness centrality topology analysis to measure the relative importance of each metabolite in a given pathway.

The relative importance of each metabolic feature in the projection used in PLS-DA models was evaluated using the Variable Importance in Projection (VIP) scores^25^. The BC *vs* CTRL model was used to screen an initial set of 128 discriminant features using VIP > 3 as a threshold. This set of metabolic features associated to the effect of TURBT in NMBIC patients, was used to build an optimized model (3 LVs), which correctly classified 29/33 BC and 11/11 CTRL samples of the validation set, providing an AUROC = 0.96 and slightly improved sensitivity (87.9 (71.8–96.6)%), specificity (100 (71.5–100)%) and the negative likelihood ratio (NLR) (0.1 (0.05–0.3)). Adjusting by the prior probability of recurrence per risk grouping at 15%, 24%, 28% and 61%, the negative predictive values for low, low-intermediate, high-intermediate and high-risk groups were 96.5%, 94.0%, 92.9% and 76.1% respectively. Figure 2 shows the VIP scores and value in the regression vector of the optimized model. Putatively identified discriminant metabolites showing a VIP > 1 in the BC *vs* CTRL model summarized in Table S1 reflected alterations in the metabolic pathways of arginine, proline, fatty acids, phenylalanine, purine, pyrimidine, and tryptophan, among others (see Table 3). Pathway analysis was used to extract biological information within relevant networks of metabolic pathways integrating metabolite set enrichment and pathway topology analysis of BC and CTRL profiles. Pathway enrichment and topology analysis were carried out using a global test and a relative betweenness centrality measure, respectively excluding unidentified or without matching HMDB ID metabolic features. Results obtained are depicted in Fig. 3, where the color and the size of each circle indicate its *p*-value and pathway impact value, respectively. Phenylalanine, arginine, proline and tryptophan pathways were found significantly altered (*p*-value < 0.05). This observation was in agreement with recent results^26^ reporting increased levels of four tryptophan metabolites (kynurenine, acetyl-N-formyl-5-methoxykynurenamine, indoleacetic acid and indolelactic acid) in serum samples of BC patients compared to healthy controls, and previous studies in BC tissue^9^ and urine^12^ that suggested the potential role of kynurenine in the malignancy BC associated to IDO and IDO2, two tryptophan-metabolizing enzymes that control the tryptophan catabolism-signaling pathway. The generation of kynurenine and other tryptophan metabolites can modulate T-cell immunity via activation of suppressive regulatory T-cells and activation of aryl hydrocarbon receptor, thus promoting cancer cell survival^27^. Higher levels of pyroglutamic acid and lower levels of hippuric acid before TURBT were in also agreement with previous studies reporting results from the analysis of urine samples collected from BC patients and reference healthy groups^28–30^. Phenylacetylglutamine is synthesized in the liver from glutamine and phenylacetyl-CoA and is a dosing biomarker for patients with urea cycle disorders; it is also a known microbial metabolite^31^. Altered levels of phenylacetylglutamine might indicate a deregulation of the phenylalanine or glutamine metabolism as well, as observed in a previous urinary metabolomic study involving BC patients and healthy controls that attributed this deregulation to the increased energy demands of cancer cells for growing and proliferation^26^. Urinary citrate is a normal component in urine and the major inhibitor of kidney stone formation. Citrate acid was found at lower concentrations before TURBT. This metabolite, key intermediate in the TCA cycle, has been repeatedly associated with an increased conversion into fatty acids required for ß-oxidation to support cancer cell proliferation. Carnitine and several carnitine metabolites were also among the most discriminant metabolites. Carnitine is an essential metabolite for the transport of long-chain fatty acids into the mitochondria and for the regulation of the intramitochondrial ratio of Acetyl-CoA to free CoA. Hence, results may support higher levels of fatty acids ß-oxidation deregulation associated to the BC tumor^11,32,33^. Altered pyrimidine and purine metabolism have been previously attributed to enhanced cancer cells cycle activity^12,34^. Taken together, the results of the present study give support to the hypothesis of the existence of a urinary metabolic profile associated with the occurrence of NMIBC tumor. Besides, the observed metabolic shift after TURBT is well aligned with such hypothesis supporting the idea of using this metabolic shift for the surveillance of cancer recurrence after TURBT in NMIBC patients.

**Table 3:** Putatively identified metabolites and associated pathways.

Pathway	Metabolites
Aminobenzoate degradation; microbial metabolism	quinone
Arginine and proline metabolism	*Creatine, creatinine*, guanidinobutanoic acid, *oxoarginine*, *gamma-glutamyl-putrescine*, *spermine*, *citrulline*
Arginine, purine, pyrimidine^12,33,34^, alanine, aspartate^33^, glutamate^32^ metabolism	*n-acetylglutamine*, thymine, *dihydrothymine*
Biosynthesis of secondary metabolites^12^	*methylxanthine*, hydroxyphenylalanine
Citrate cycle^11,12,29,34,35^	*citric acid* ^12,29,34^
Energy metabolism	*carnitine*^35^, acetylcarnitine^30^, *o-isobutyryl-carnitine*, *3-methylglutarylcarnitine, propionylcarnitine*
Fatty acid metabolism^30,33,35^	*carnitine*^35^, *furoylglycine*, aminohippuric acid, hydroxyhippuric acid
Glutathione metabolism	pyroglutamic acid
Phenylalanine metabolism^30^	hydroxyhippuric acid, *hippuric acid*^29,30,36^, phenylacetylglutamine^30^, *phenyllactic*, *hydrocinnamic acid*, homophenylalanine, *phenylacetylglycine*, *aminosalicyluric acid*, phenylglyoxylic acid, tyrosine^32^
Primary degradation product of tRNA	*dimethylguanosine*
Purine metabolism^11,32,33^	*hypoxanthine*^32^, *methylhypoxanthine*, *adenosine*^8^, xanthine, uric acid^32^
Tryptophan metabolism^12,32,35^	tyrosine^32^, hydroxyindole, *hydroxyanthranilic acid*, anthranilic acid^12^, indolelactic acid, *methyltryptamine*, tryptophan^32^, *hydroxyindoleacetic acid*, kynurenine, *hydroxyindolepyruvic acid*, hydroxytryptophan

References indicate previous clinical urinary metabolomic studies of BC in which the metabolite were selected as discriminant and/or dysregulated pathways reviewed in^15^. Note: Metabolites found at higher levels before TURBT are highlighted in bold. Pathways highlighted in bold were found disregulated (*p*-value < 0.05) (see the text for details).

### Longitudinal analysis of metabolic changes during surveillance for recurrence

**Figure 4:**
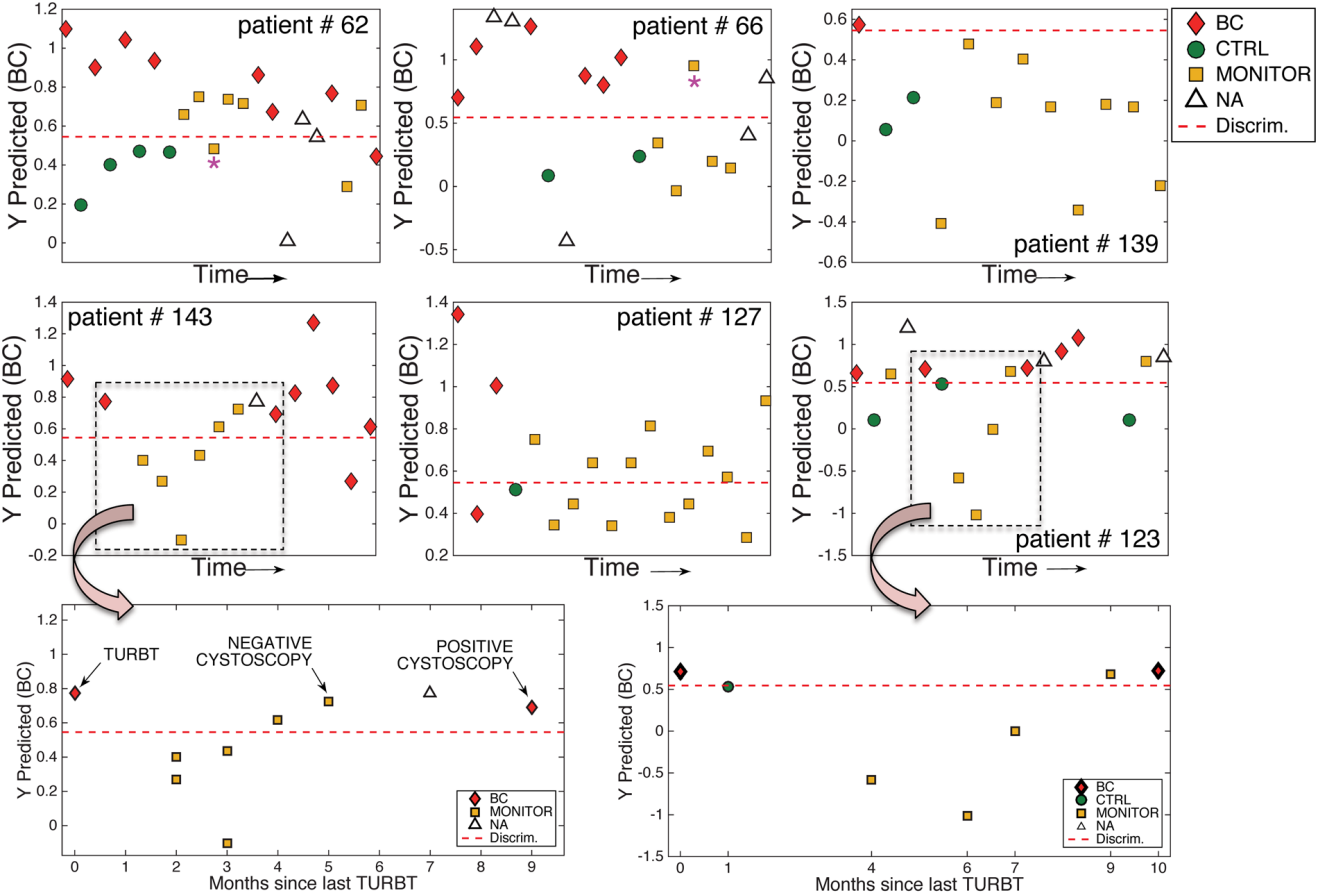
Analysis of longitudinal trajectories after TURBT. Predicted *y* PLS-DA values in 6 patients during surveillance of BC recurrence. Note: (*) indicates a MONITOR sample showing an inconsistent trajectory with a gradual progression of the disease after TURBT. BC and CTRL samples from patients 66 and 123 were included in the train set.

The analysis of the longitudinal trajectories of the metabolic biomarkers discriminating between BC and CTRL samples, allowed a preliminary evaluation of its potential utility to monitor NMIBC relapse in patients undergoing surveillance for tumor recurrence. Figure 4 shows the predicted *y* PLS-DA values in 6 patients with multiple episodes of BC recurrence. Results show CTRL and BC samples were accurately classified (90.1% overall accuracy in the test set) and, for some patients (see patients #123 and #143), the longitudinal trajectory during surveillance indicated a gradual shift of the metabolic profile towards a BC profile, that was consistent with the confirmatory results of cystoscopy and, especially in the case of #143, seemed to anticipate results obtained by cystoscopy. Figure 4 also depicts results from the analysis of samples collected during negative cancer recurrence surveillance (see patients #66, #127 and #139). In these cases, no clear trend that could be associated with a BC progression was observed after TURBT, also consistent with the clinical observations.

Nevertheless, in few patients, the follow up of biomarkers showed trajectories inconsistent with a gradual progression of the disease after TURBT (see e.g. MONITOR samples from patients #62 and 66 marked by an * in Fig. 4). The fact that urine samples can be easily obtained from patients, and that urine is in close contact with the tumor cells in NMIBC patients, are two *a priori* advantageous features for the development of a non-invasive metabolomic analysis. However, urine analysis is challenging due to the variation in chemical composition and concentrations across and within individuals. A wide range of potential confounding factors such as individual genotype, diet, water consumption, environmental exposure or drug intake may affect the urine metabolome. The effect of uncontrolled sources of variation may justify the abovementioned few anomalous observations in the test set. We minimized such effects by comparing urine samples before and after TURBT, and after tumor relapse. However, further research is needed to assess the sources of variability in urine and increase the robustness of metabolic tests in exploratory studies to facilitate the validation and translation of biomarker discoveries into clinical practice.

## Conclusions

Results from this exploratory clinical study disclosed a statistically significant shift in the urinary metabolic profile in NMIBC patients before and after TURBT and give support to the hypothesis of a specific urinary metabolic profile associated to the occurrence of an NMIBC tumor. Consistent with this view, follow up of the urine metabolome in the course of cancer recurrence surveillance revealed a gradual shift in the metabolic profile towards the BC profile when the tumor reappeared, as confirmed by cystoscopy. Taken together, these results provide a strong basis for the use of a metabolomic-based biomarker analysis as a non-invasive monitoring system to detect NMIBC recurrence at an early stage and eventually adjust therapies according to NMIBC risk. A larger sample size of representative samples towards the size of a population should, theoretically, lead to more generalizable results and increase the ability to discriminate patients pre- and post-resection. Larger population sizes need to be studied during the clinical validation of the proposed biomarkers. Moreover, a better characterization of the differences between pre- and post-resection urinary metabolic profiles may result in improved models with larger effect sizes.

## Electronic supplementary material

Supplementary Information
