# Supplementary material for: Bladder cancer recurrence surveillance by urine metabolomics analysis
Source: Sci Rep. 2018 Jun 15;8:9172. doi: 10.1038/s41598-018-27538-3 (PMC6004013; doi:10.1038/s41598-018-27538-3)
Supplement: Supplementary file 1 — Supplementary Information [file 41598_2018_27538_MOESM1_ESM.pdf]

Bladder cancer recurrence surveillance by urine metabolomics analysis

A. Loras[a], M. Trassierra[b]*, D. Sanjuan-Herráez[c], M.C. Martínez-Bisbal[a], J.V. Castell[d,e], G. Quintás[c,f]**, J.L. Ruiz-Cerdá[a,b]*

[a]Unidad Mixta de Investigación en Nanomedicina y Sensores, Universidad Politécnica de Valencia – IIS La Fe, Valencia, Spain

[b]Urology Service, Hospital Universitario y Politécnico La Fe, Valencia, Spain

[c]Health & Biomedicine, Leitat Technological Center, Barcelona, Spain

[d]Departamento de Bioquímica y Biología Molecular, Universidad de Valencia, Valencia, Spain

[e]Unidad de Hepatología Experimental, Health Research Institute La Fe, Valencia, Spain

[f]Analytical Unit, Health Research Institute La Fe, Valencia, Spain

** contributed equally to this work.*

***e-mail: gquintas@leitat.org*

SUPPORTING INFORMATION

Figure S-1. Quality control of instrument performance and metabolomic data pretreatment. A) Variation of the m/z accuracy and RT of a spiked internal standard (Caffeine-D_9) as a function of the injection order; B) peak area values of five selected metabolites: phenylalanine (Phe), tryptophan (Trp), kynurenine (Kyn), hydroxykynurenine (OHKyn) and phenylacetylglutamine (PAGN) using manual (MassHunter) and automated (XCMS) integration; C) intensity of Peak area values of five selected metabolites as a function of the injection order in raw data; D) intensity of Peak area values of five selected metabolites as a function of the injection order after batch effect correction.

Figure S-2. Distribution of RSD(QCs)% in batches 1 and 2 (left) and PCA scores as a function of injection order (right) in raw data (top) and after batch effect correction (bottom).

Figure S-3. PCA scores plot using the set of QC, BC, CTRL and MONITOR samples after MSC and pareto scaling as data pretreatment.

Table S-1. Urinary metabolites putatively identified among the set of metabolic features with VIP>1 in the initial BC *vs* CTRL PLS-DA model. Note: t-test: t-test *p*-value; BC/CTRL: ratio mean value in BC over CTRL samples.

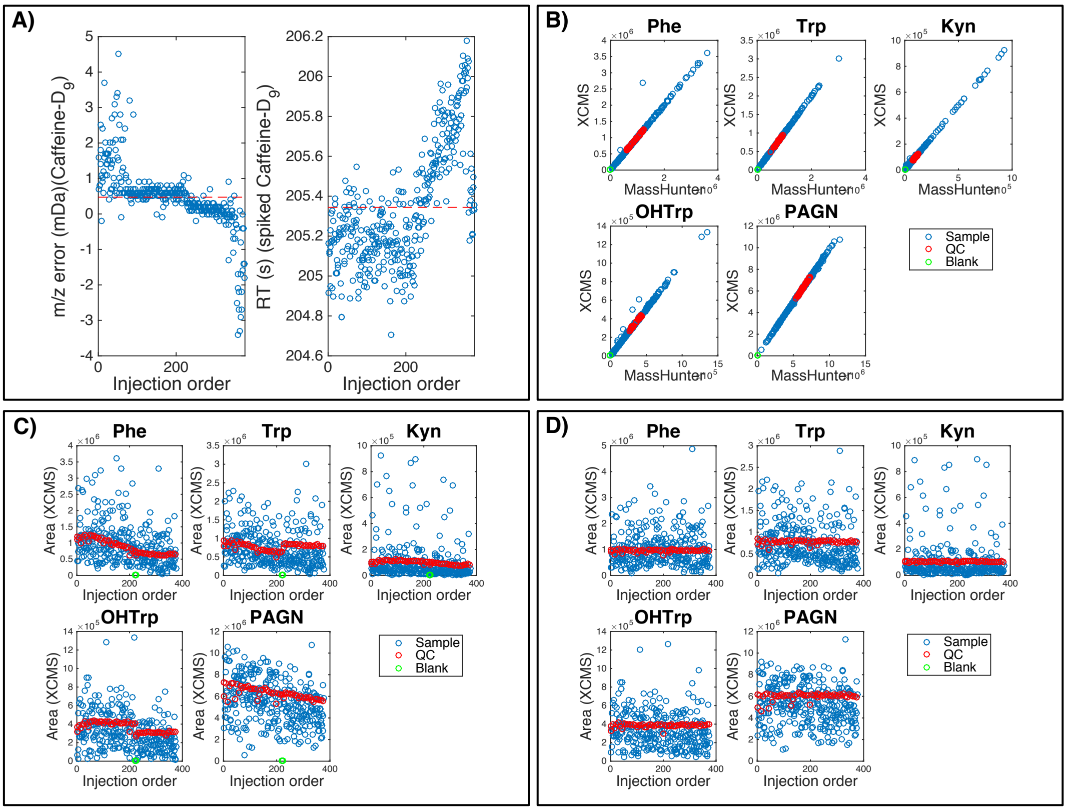

Figure S-1. Quality control of instrument performance and metabolomic data pretreatment. A) Variation of the m/z accuracy and RT of a spiked internal standard (Caffeine-D9) as a function of the injection order; B) peak area values of five selected metabolites: phenylalanine (Phe), tryptophan (Trp), kynurenine (Kyn), hydroxykynurenine (OHKyn) and phenylacetylglutamine (PAGN) using manual (MassHunter) and automated (XCMS) integration; C) intensity of Peak area values of five selected metabolites as a function of the injection order in raw data; D) intensity of Peak area values of five selected metabolites as a function of the injection order after batch effect correction.

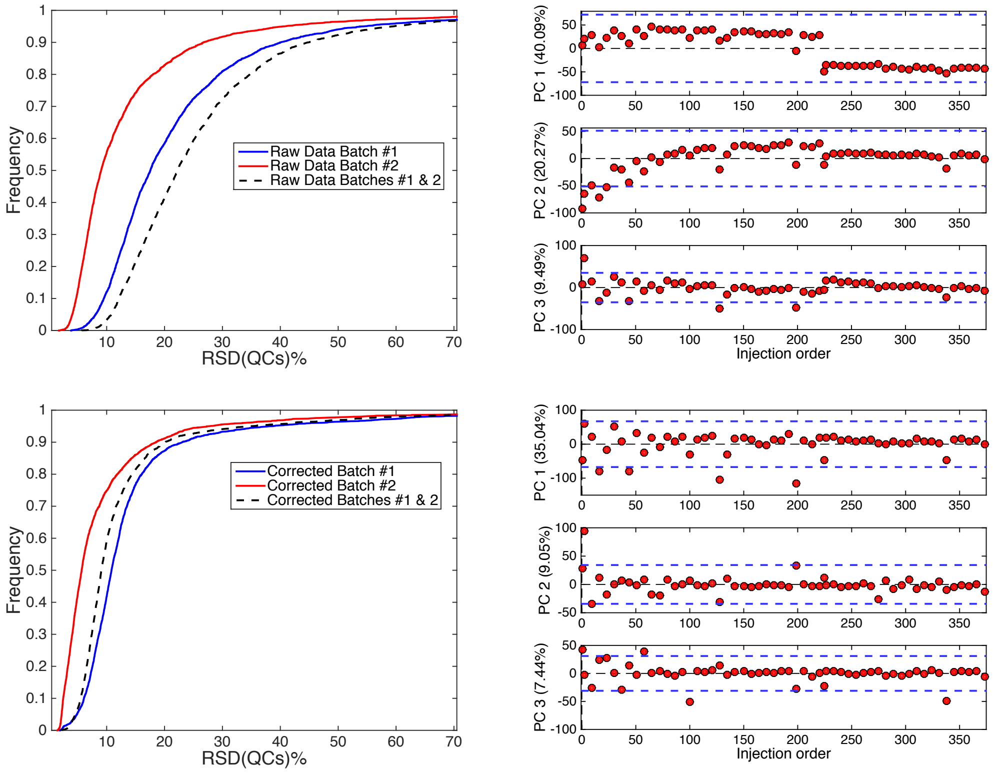

Figure S-2. Distribution of RSD(QCs)% in batches 1 and 2 (left) and PCA scores as a function of injection order (right) in raw data (top) and after batch effect correction (bottom).

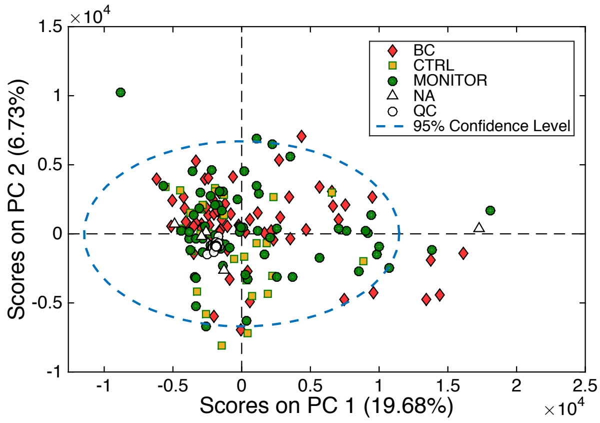

Figure S-3. PCA scores plot using the set of QC, BC, CTRL and MONITOR samples after MSC and pareto scaling as data pretreatment.

Table S1. Urinary metabolites putatively identified among the set of metabolic features with VIP>1 in the initial BC *vs* CTRL PLSDA model. Note: t-test: t-test *p*-value; BC/CTRL: ratio mean value in BC over CTRL samples.

ID	Mol. Fmla.	Error (ppm)	Score	Putative ID	HMDB/KEGG	t-test	BC/CTRL
109.0285-3.37	[C6H5O2]+	-3	99	Quinone	HMDB03364	<0.05	>1
115.0693-0.67	[C4H8N3O]+	2	98	Creatinine	HMDB00562	>0.05	<1
146.0921-0.83	[C5H12N3O3]+	2	90	Guanidinobutanoic	HMDB03464	>0.05	>1
174.0873-0.76	[C6H12N3O3]+	0.9	85	Oxoarginine	HMDB04225	>0.05	<1
182.1291-1.12	[C9H16N3O]+	0.9	87	g-glutamyl-putrescine	HMDB12230	<0.05	<1
203.2231-052	[C10H27N4]+	-1	87	Spermine	HMDB01256	<0.05	<1
176.1033-0.78	[C6H14N3O3]+	0.1	90	Citrulline	HMDB00904	>0.05	>1
171.0761-0.70	[C7H11N2O3]+	-3	83	Acetylglutamine	HMDB06029	>0.05	<1
127.0488-1.95	[C5H7N2O2]+	-0.1	NA	Thymine	HMDB00262	<0.05	>1
129.0655-0.67	[C5H9N2O2]+	-3	87	Hydrouracil	HMDB00079	<0.05	<1
167.0567-1.77	[C6H7N4O2]+	-4	92	Methylxanthine	HMDB01991	>0.05	<1
182.0817-3.89	[C9H12NO3]+	-4	82	Hydroxy-phenylalanine	C19712	<0.05	>1
215.0167-0.86	[C6H8O7Na]+	-5	85	Citric acid	HMDB00094	>0.05	<1
220.0642 -1.55	[C11H11NO4]+	-6	83	Hydroxyindolepyruvate	C05646	>0.05	<1
162.1123-0.66	[C7H16NO3]+	0.6	82	Carnitine	HMDB00062	>0.05	<1
204.1239-0.91	[C9H18NO4]+	-8	86	Acetylcarnitine	HMDB00201	>0.05	<1
232.1551-2.75	[C11H22NO4]+	-3	93	Isobutyryl carnitine	HMDB62606	>0.05	<1
290.1613-2.50	[C13H24NO6]+	-5	92	Methylglutarylcarnitine	HMDB00052	>0.05	<1
170.0452-2.19	[C7H8NO4]+	-5	93	Furoylglycine	HMDB00439	<0.05	>1
245.0928-5.19	[C11H14N2O3Na]+	-3	95	Aminohippuric acid	HMDB01867	<0.05	>1
218.0430-2.29	[C9H9NO4Na]+	-4	86	Hydroxyhippuric acid	HMDB06116	>0.05	<1
180.0660-3.51	[C9H10NO3]+	-4	97	Hippuric acid	HMDB00714	<0.05	<1
265.1185-3.67	[C13H17N2O4]+	1	98	Phenylacetylglutamine	HMDB06344	>0.05	<1
130.0506-1.31	[C5H8NO3]+	6	97	Pyroglutamic acid	HMDB00267	>0.05	>1
151.0753-5.60	[C9H11O2]+	-2	86	Hydrocinnamic acid	HMDB00764	>0.05	>1
180.1017-6.09	[C10H14NO2]+	0.1	88	Homophenylalanine	NA	<0.05	<1
194.0812-4.04	[C10H11NO3]+	0.9	75	Phenylacetylglycine	HMDB00821	<0.05	<1
211.0718-1.90	[C9H11N2O4]+	-2	97	Aminosalicyluric acid	HMDB61683	>0.05	<1
151.0393-2.85	[C8H7O3]+	-4	96	Phenylglyoxylic acid	HMDB60026	<0.05	>1
182.0790-2.23	[C9H12NO3]+	-4	95	Tyrosine	HMDB00158	>0.05	>1
312.1306-2.44	[C12H18N5O5]+	-3	96	Dimethylguanosine	HMDB04824	>0.05	<1
137.0460-0.88	[C5H5N4O]+	-3	91	Hypoxanthine	HMDB00157	>0.05	>1
151.0618-1.89	[C6H7N4O]+	-4	88	Methylhypoxanthine	HMDB13141	>0.05	<1
268.1044-1.07	[C10H14B5O4]+	-2	91	Adenosine	HMDB00050	>0.05	<1
153.0407-0.89	[C5H4N4O2]+	-6	85	Xanthine	HMDB00292	>0.05	>1
169.0360-0.80	[C5H4N5O3]+	-5	82	Uric acid	HMDB00289	>0.05	>1

Table S1. Urinary metabolites putatively identified among the set of metabolic features with VIP>1 in the initial BC *vs* CTRL PLSDA model. Note: t-test: t-test *p*-value; BC/CTRL: ratio mean value in BC over CTRL samples.

ID	Mol. Fmla.	E (ppm)	Score	Putative ID	HMDB/KEGG	t-test	BC/CTRL
134.0600-3.33	[C8H8NO]+	-2	87	Hydroxyindole	HMDB59805	>0.05	>1
154.0501-1.13	[C7H8NO3]+	-4	83	Hydroxyanthranilic	HMDB01476	>0.05	<1
138.0549-0.67	[C7H8NO2]+	-6	94	Anhranillic acid	HMDB01123	>0.05	>1
175.1237-2.65	[C11H15N2]+	-5	93	Methyltryptamine	HMDB04370	<0.05	<1
205.0980-2.86	[C11H13N2O2]+	-5	96	Tryptophan	HMDB00929	>0.05	>1
192.0659-1.99	[C10H10NO3]+	-3	85	Hydroxyindoleacetic acid	HMDB00763	>0.05	<1
209.0925-1.99	[C10H13N2O3]+	-2	87	Kynurenine	HMDB00684	>0.05	>1
221.0925-1.50	[C11H13N2O3]+	-3	83	Hydroxytryptophan	HMDB00472	>0.05	>1
132.0765-0.68	[C6H14N2O]+	-2	86	Creatine	HMDB00687	>0.05	>1
200.1281-2.41	[C10H18NO3]+	-4	82	Propionylcarnitine	HMDB00824	<0.05	>1
129.0655-0.67	[C5H9N2O2]+	-2	87	Dihydrothymine	HMDB00079	<0.05	<1
126.0657-0.88	[C5H8N3O]+	-6	83	Methylcytosine	HMDB02894	<0.05	<1
